# Crystal structure of Sec10, a subunit of the exocyst complex

**DOI:** 10.1038/srep40909

**Published:** 2017-01-18

**Authors:** Jianxing Chen, Atsushi Yamagata, Keiko Kubota, Yusuke Sato, Sakurako Goto-Ito, Shuya Fukai

**Affiliations:** 1 Structural Biology Laboratory, Structural Life Science Division, Synchrotron Radiation Research Organization and Institute of Molecular and Cellular Biosciences, The University of Tokyo, Tokyo 113-0032, Japan; 2 Department of Computational Biology and Medical Sciences, Graduate School of Frontier Sciences, The University of Tokyo, Chiba 277-8501, Japan; 3 CREST, JST, Saitama 332-0012, Japan

## Abstract

The exocyst complex is a heterooctameric protein complex composed of Sec3, Sec5, Sec6, Sec8, Sec10, Sec15, Exo70 and Exo84. This complex plays an essential role in trafficking secretory vesicles to the plasma membrane through its interaction with phosphatidylinositol 4,5-bisphosphate and small GTPases. To date, the near-full-length structural information of each subunit has been limited to Exo70, although the C-terminal half structures of Sec6, Sec15 and Exo84 and the structures of the small GTPase-binding domains of Sec3, Sec5 and Exo84 have been reported. Here, we report the crystal structure of the near-full-length zebrafish Sec10 (zSec10) at 2.73 Å resolution. The structure of zSec10 consists of tandem antiparallel helix bundles that form a straight rod, like helical core regions of other exocyst subunits. This structure provides the first atomic details of Sec10, which may be useful for future functional and structural studies of this subunit and the exocyst complex.

Exocytosis is a fundamental cellular process that mediates secretion of biologically active molecules to the extracellular environment and transport of lipids and membrane proteins to the plasma membrane in eukaryotic cells^1^. This process occurs with fusion between secretory vesicles and the plasma membrane. In the initial step of the fusion, it has been postulated that the vesicles and the plasma membrane are physically and reversibly connected through a heterooctameric protein complex termed the exocyst complex^2^. The exocyst complex is required for trafficking of secretory vesicles to the plasma membrane^3^,^4^,^5^. The disruption of its function causes defects in various biological processes including cell polarity^6^, cell migration^7^, primary ciliogenesis^8^, neurite outgrowth^9^ and autophagy^10^. The initial connection between trafficking vesicles and their target membranes has also been postulated in other intracellular trafficking pathways and is traditionally called tethering. The tethering process may accelerate capture of the specific vesicles and/or the following SNARE complex assembly for catalyzing the membrane fusion^11^. The exocyst complex belongs to a family of evolutionally conserved complexes associated with tethering containing helical rods (CATCHR), which includes other multisubunit tethering complexes (MTC) such as conserved oligomeric Golgi (COG), Dsl1 and Golgi-associated retrograde protein (GARP) complexes^12^.

The exocyst complex consists of Sec3, Sec5, Sec6, Sec8, Sec10, Sec15, Exo70 and Exo84. The genes encoding Sec3, Sec5, Sec6, Sec8, Sec10 and Sec15 were first isolated from temperature-sensitive secretory-deficient (*sec*) mutants of budding yeast^3^. Then, the other two subunits Exo70 and Exo84 were biochemically identified from the exocyst complex purified from yeast and rat, respectively^4^,^5^. To date, three-dimensional structures of near-full-length or partial forms of Sec3, Sec5, Sec6, Sec15, Exo70 and Exo84 have been reported: yeast Sec3_75–241_ and Sec3_75–260_, rat Sec5_4–95_, yeast Sec6_411–805_, fruitfly Sec15_383–699_, yeast Exo70_67–623_, mouse Exo70_85–653_, thale cress Exo70_75–629_, yeast Exo84_525–753_ and rat Exo84_171–279_^13^,^14^,^15^,^16^,^17^,^18^,^19^,^20^,^21^. Core helical regions of Sec6, Sec15, Exo70 and Exo84 exhibit similar rodlike structures, which are composed of consecutively packed α-helix bundles with a characteristic α-solenoid topology. Bioinformatics analysis has predicted that all exocyst subunits contain similar rodlike structures^22^, although three-dimensional structures of the core helical regions of Sec3, Sec5, Sec8 and Sec10 remain unknown. Structures of other CATCHR subunits also adopt the conserved α-solenoid topology, suggesting that the CATCHR family members have diverged from a common ancestor to become specialized in their specific trafficking pathways^12^.

Pairwise interactions between the individual exocyst subunits have been investigated by pull-down and yeast two-hybrid assays (summarized in ref. 22). Furthermore, two recent studies that monitored the subunit assembly occurring in cells suggest that the exocyst complex can be divided into two assembly modules: one contains Sec3, Sec5, Sec6 and Sec8, whereas the other contains Sec10, Sec15, Exo70 and Exo84^23^,^24^. Sec10 appears to serve as a central hub for bridging these two assembly modules, possibly through its interaction with Sec6 and/or Sec8^23^,^24^. The functional importance of Sec10 has been assessed by enhancement or suppression of Sec10 at the cellular level^9^,^25^,^26^. Overexpression of Sec10 increases the exocyst-mediated vesicular trafficking to the bud in yeast and to the basolateral surface in Madin-Darby canine kidney cells. Conversely, overexpression of dominant-negative Sec10 or knockdown or knockout of Sec10 causes morphological defects in budding yeast or inhibition of neurite outgrowth in neurons. In mammalian cells, Sec10 interacts with small GTPase Arf6, which can restrict clathrin-mediated endocytosis sites to the apical surface of epithelial cells^27^,^28^,^29^. Sec10 is also involved in the primary ciliogenesis on the apical surface^30^, besides targeting trafficking vesicles to the basolateral surface of epithelial cells.

Despite the importance of Sec10 in exocyst-mediated cellular processes, three-dimensional structural information of Sec10 remains unknown. In this study, we report the near-full-length structure of zebrafish Sec10 (zSec10). This structure provides the first atomic details of this exocyst subunit.

## Results

### Overall structure

**Figure 1:**
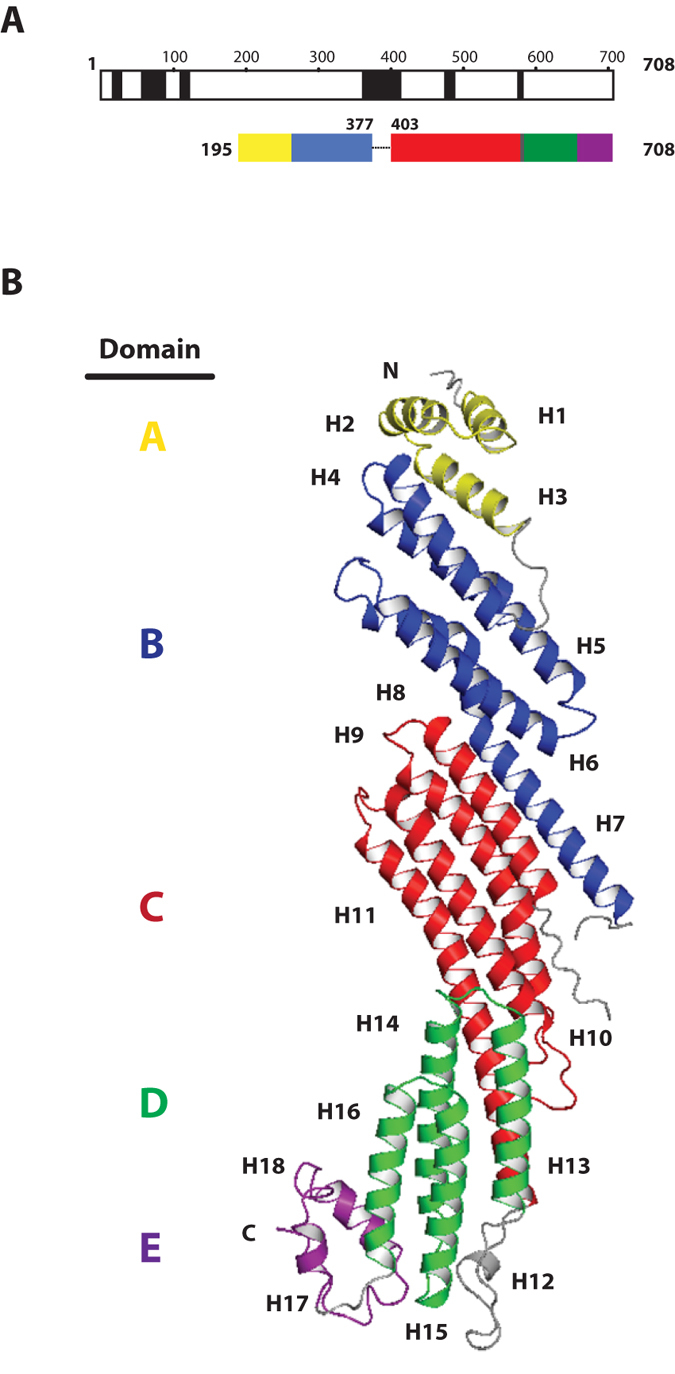
Overall structure of zSec10_195–708_ (Δ385–394). (**A**) Schematic figures representing the predicted disordered regions (residues 23–37, 57–89, 115–128, 365–412, 468–480 and 565–569 in black, top) and domain architecture of zSec10_195–708_ (Δ385–394) (bottom), residues 378–384 and 395–402 are invisible in the structure. The domain boundaries were defined on the basis of the crystal structure of zSec10_195–708_ (Δ385–394). Domains (A–E) are in yellow, blue, red, green and purple, respectively. (**B**) Crystal structure of zSec10_195–708_ (Δ385–394). The coloring scheme of each domain is the same as that in (**A**).

The recombinant full-length zSec10 (residues 1–708) tended to aggregate during purification. This property is similar to that of rat Sec10 (rSec10), from which we failed to obtain high-resolution crystals. It was assumed that the aggregation properties of full-length zSec10 and rSec10 might be due to partial structural disorders, which were predicted computationally using the program DISOPRED2^31^ (Fig. 1A) and experimentally by limited proteolytic analyses. On the basis of the information on the computationally and experimentally predicted disordered regions, we designed sets of expression constructs of rSec10 and zSec10 (Supplementary Table 1) and tested their expression in *Escherichia coli*, purification and crystallization. Basically, removal of the major predicted disordered regions enhanced the expression of soluble rSec10 and zSec10 in *E. coli* and improved their stability during purification. Among the expression constructs tested in this study, we obtained clusters of daggerlike small crystals from zSec10_195–708_ (Δ385–394). For data collection, single crystals of the native or selenomethionine (SeMet)-substituted zSec10 were grown by streak seeding with crushed native crystals as the seed. Bromide soaking improved the diffraction from ~4 Å to 3 Å or higher. The structure of zSec10_195–708_ (Δ385–394) was determined by a single-wavelength anomalous dispersion (SAD) method and refined to 2.73 Å. The crystals contain one protein molecule per asymmetric unit. Residues 378–384 and 395–402 were invisible, probably owing to the structural disorder. The final refined model includes 10 bromide atoms and 2 water molecules. The bound bromide atoms were confirmed using an anomalous difference Fourier map, although their anomalous signals were insufficient for phasing. Data collection and refinement statistics are shown in Table 1.

**Figure 2:**
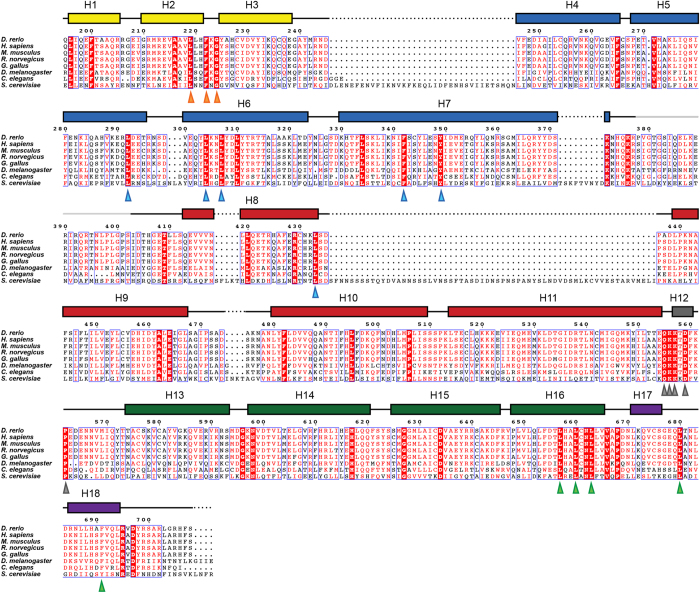
Amino-acid sequence alignment of Sec10. Amino-acid sequence alignment of Sec10 from 8 representative organisms (*Danio rerio, Homo sapiens, Mus musculus, Rattus norvegicus, Gallus gallus, Drosophila melanogaster, Caenorhabditis elegans* and *Saccharomyces cerevisiae*). The invariant residues are indicated as white characters with a red background, whereas the conserved residues are indicated as red characters. The α-helical regions are represented as boxes above the alignment. The coloring scheme is based on the domain assignment shown in Fig. 1B. The residues comprising the four main conserved regions (i)–(iv) are indicated by orange, cyan, grey and light green triangles, respectively.

**Figure 3:**
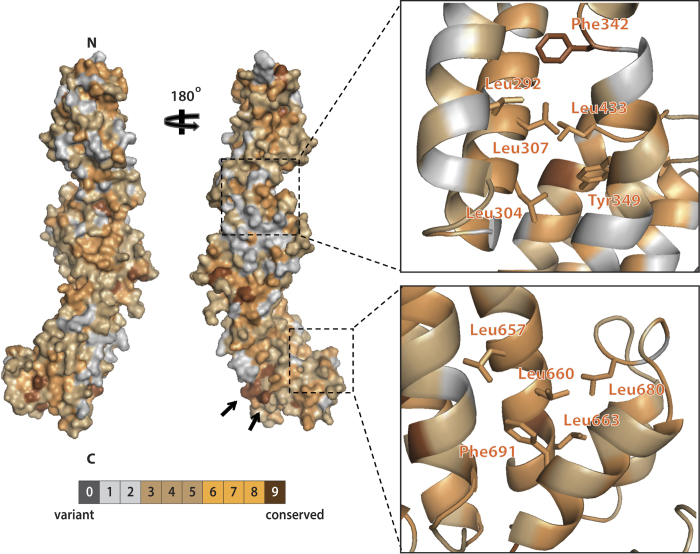
Residue conservation of zSec10. Surface representation that shows the residue conservation of zSec10. The level of residue conservation is indicated as a brown gradient. Two black arrows indicate the conserved Lys556 and Lys557 exposed in the C-terminal region. The close-up views show the conserved hydrophobic cores that stabilize the helix bundle composed of helices H6, H7 and H8 (top) and the relative conformation between domains D and E (bottom).

zSec10 folds into an elongated rod with dimensions of 150 Å × 40 Å × 25 Å. The structure of zSec10 can be divided into five domains A–E, each of which is composed of an antiparallel helix bundle. Domains A, B–D and E contain three (H1–H3), four (H4–H7, H8–H11, H13–H16) and two (H17–H18) helices, respectively (Fig. 1B). These domains are connected by long solvent-exposed loops (residues 373–412 connecting domains B and C, and residues 555–573 connecting domains C and D) or short turns (residues 239–245 connecting domains A and B, and residues 666–670 connecting domains D and E). Helix H7 is shared between domains B and C, whereas helix H11 is shared between domains C and D. These two long helices serve as the bridge connecting two adjoining subdomains. Similar bridge helices have also been found in other CATCHR subunits, indicating a common mechanism of building a long rodlike protein from individual helix bundles in the CATCHR subunits. The Sec10 residues that are highly conserved among representative species are located mainly in four regions: (i) the loop connecting helices H2 and H3, (ii) the hydrophobic core stabilizing the helix bundle composed of helices H6, H7 and H8, (iii) helix H12 located within the long loop between domains C and D, and (iv) the hydrophobic core maintaining the relative spatial arrangement of domains D and E (Figs 2 and 3). Most of them are hydrophobic residues that are buried inside the protein.

In our limited proteolytic experiment of zSec10, tryptic cleavage occurred at Arg394 and generated two polypeptides corresponding to the N- and C-terminal halves. These two polypeptides were co-eluted as a single peak in gel-filtration chromatography (Supplementary Fig. 1A), indicating that the N- and C-terminal halves of Sec10 can interact with each other, even without the linker connecting them. The hydrogen bond between the conserved Glu353 in helix H7 and Arg429 in helix H8 may contribute to this interdomain interaction, together with the nearby hydrophobic residues (Supplementary Fig. 1B).

### Comparison with CATCHR subunits and their structurally related proteins

**Figure 4:**
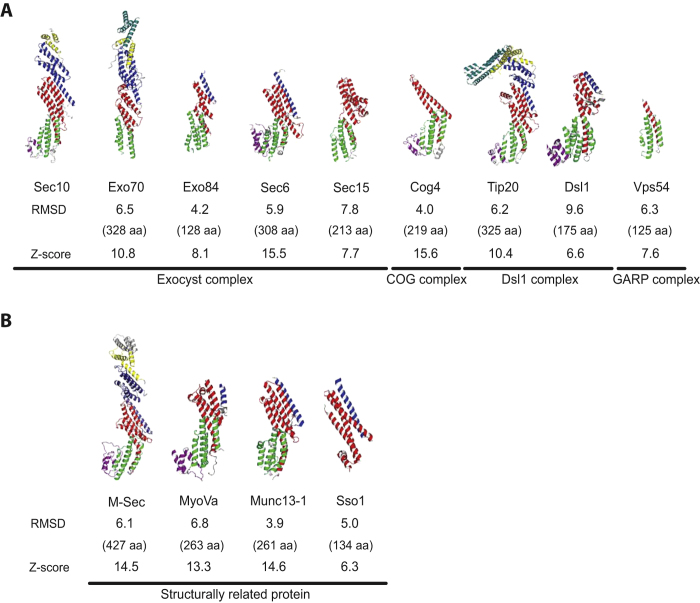
Structural comparison of zSec10 with other CATCHR subunits and their structurally related proteins. The rmsd and Z-score calculated using the Dali server are shown below each structure. The lengths of alignment for calculating rmsd are shown in parentheses. (**A**) Structural comparison of zSec10 with other CATCHR subunits (*A. thaliana* Exo70 [PDB 4RL5], *S. cerevisiae* Exo84 [PDB 2D2S], *S. cerevisiae* Sec6 [PDB 2 FJI], *D. melanogaster* Sec15 [PDB 2A2F], *H. sapiens* Cog4 [PDB 3HR0], *S. cerevisiae* Tip20 [PDB 3FHN], *K. lactis* Dsl1 [PDB 3K8P] and *M. musculus* Vps54 [PDB 3N1B]). (**B**) Structural comparison of zSec10 with its structurally related proteins (*M. musculus* M-Sec [PDB 5B86], *H. sapiens* myosin Va [PDB 4LLI], *R. norvegicus* Munc13–1 [PDB 3SWH] and *S. cerevisiae* Sso1 [PDB 1FIO]).

**Figure 5:**
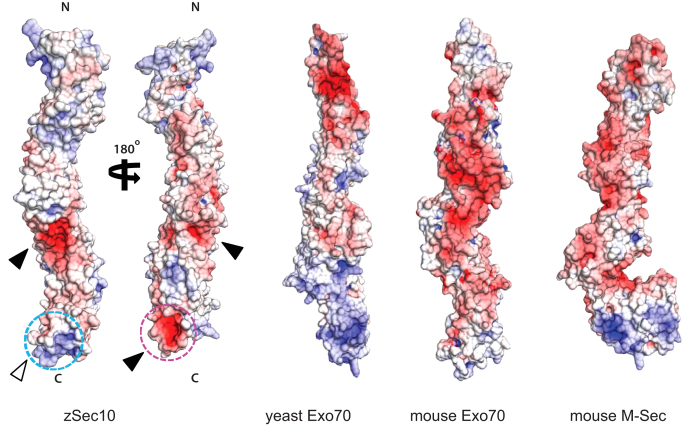
Electrostatic surface potential distribution of zSec10. Electrostatic surface potential of zSec10, yeast Exo70, mouse Exo70 and mouse M-Sec on a scale from −10 *k*_B_*T/e* (red) to +10 *k*_B_*T/e* (blue). Black and white arrows indicate negatively and positively charged patches in the C-terminal half of zSec10, respectively. The potential interaction site for Arf6 and the conserved positively charged patch in the C-terminal end are encircled by purple and cyan dotted lines, respectively.

The crystal structure of zSec10_195–708_ (Δ385–394) bears an expectedly strong resemblance in helical topology to other subunits of the exocyst complex and other CATCHR subunits^16^,^17^,^19^,^21^,^32^,^33^,^34^ with Z-scores of 6.6–15.6 and rmsd values of 4.0–9.6 Å (calculated by the Dali server^35^) (Fig. 4A). A similar topology is also shared with the Sec6-homologous protein M-Sec, the globular tail domain of the myosin V family, the MUN domain of the Munc13 family and the t-SNARE protein Sso1^36^,^37^,^38^,^39^ with Z-scores of 6.3–14.6 and rmsd values of 3.9–6.8 (Fig. 4B). Among these proteins, the near-full-length structures of only Exo70, M-Sec and Tip20 have been determined. Sec10 exhibits a straight rod structure similarly to Exo70 and M-Sec, but differs in shape from Tip20, which adopts a sharply hooked structure at the junction between domains B and C (Fig. 4). The N-terminal half of yeast Exo70 exhibits a strongly negative charge, whereas the C-terminal half a positive charge. On the other hand, mouse Exo70 is negatively charged overall^18^. Mouse M-Sec is also negatively charged overall, except for the positively charged C-terminal end^36^ (Fig. 5). In contrast, zSec10 shows no substantial charge polarity on its surface, but several negatively or positively charged pockets were observed in its C-terminal half (Fig. 5). The zSec10 structure, as well as the Tip20 and M-Sec structures, shows no biased distribution of hydrophobicity on its surface, although the C-terminal half of yeast Sec10 was predicted to be more hydrophobic than the N-terminal half^26^ (Supplementary Fig. 3).

For other CATCHR subunits, the structures of their C-terminal halves have been reported (Fig. 4A). Structural classification using the Dali server^35^ indicated that Sec10 is most similar to Cog4 with the best Z-score of 15.6 and rmsd value of 4.0 Å (Fig. 4A) among all CATCHR subunits and their structurally related proteins of known structures. Pairwise superposition of Sec10 onto other CATCHR subunits (Sec6, Cog4, Tip20, Dsl1, M-Sec and MyoVa) highlights the remarkable structural conservation of domain D with an average rmsd value of 2.1 Å; domains C and E seem more divergent than domain D with average rmsd values of 3.5 and 3.2 Å, respectively (Cog4 was excluded for domain C, because two helices are missing in the domain C structure of Cog4) (Supplementary Fig. 2).

### Common functional domain D

Domain D of the CATCHR subunit is functionally important. For example, the Rho3-binding site of Exo70 and the Rab11-binding site of Sec15 are located within the third helix of domain D, which corresponds to helix H15 in zSec10^17^,^40^ (Supplementary Fig. 2). Mutations of Sec6 that cause mislocalization of the exocyst complex are positioned in the third helix (residues 624–645) of domain D in Sec6, which corresponds to helix H13 in zSec10^41^. A conserved positively charged patch in M-Sec is located in the short turn between the helices corresponding to helices H14 and H15 of zSec10^36^. Mutations in the positively charged patch eliminated the M-Sec-induced membrane protrusion. In Sec6, Cog4, Tip20 and M-Sec, domain E is additionally flanked by domain D (Fig. 4). Domains D and E of Cog4 interact with each other through the electrostatic interaction between the conserved arginine and glutamate residues. Mutations disturbing this interaction cause cell surface glycosylation defects^32^.

In yeast, the C-terminal half of Sec10 is required for its tethering function, whereas the N-terminal half of Sec10 has been suggested to engage in the assembly of the exocyst complex^26^. The C-terminal half (*i.e.*, domains C–E) of Sec10 reportedly interacts with Arf6^27^. In the zSec10 structure, a conserved hydrophobic interaction stabilizes the relative conformation between domains D and E, as mentioned above. Deletion of the C-terminal region of rSec10 (residues 606–708), including most parts of domain D and the entire domain E, prevents the association of rSec10 with Arf6, as shown by yeast two-hybrid assay^27^. The corresponding region of zSec10 forms a cave with negative charges, which are derived primarily from acidic residues on the loop connecting domains C and D. This cave could electrostatically interact with the conserved lysine residues (*i.e.*, Lys3, Lys7 and Lys12) of Arf6 in the N-terminal region, which is required for the interaction with Sec10^27^. In addition, the conserved positively charged patch (Fig. 5) located at the C-terminus of domain E potentially interacts with the negatively charged phosphoinositides in the membrane, in analogy to yeast Exo70, which interacts with PI(4,5)P_2_ and Rho3 *via* the C-terminal region^19^.

## Discussion

To date, the N-terminal half structures of CATCHR subunits and their structurally related proteins have remained mostly unknown. The present zSec10 structure is the second near-full-length structure of the exocyst subunits determined so far. Yeast, mouse or thale cress Exo70 could be crystalized with small N- and C-terminal truncations, whereas the crystallization of zSec10 additionally required the truncation of the middle region. In this study, we combined disorder prediction and limited proteolysis to identify the middle region whose truncation could enhance the protein solubility and optimize the expression construct for crystallographic studies (Supplementary Table 1). It has been predicted that most of the CATCHR subunits might consist of similar helical architectures, where the N- and C-terminal half structural units are connected by the middle linker region^22^. Truncation of the middle region could be generally applied to crystallographic or other structural studies of other CATCHR subunits of near-full-length forms. For this strategy, we need to pay attention to maintaining the topology of the protein structure and intramolecular interaction, because shortening or deleting a flexible linker region may affect them in some cases^42^. zSec10_195–708_ (Δ385–394) retains ~75% of its unstructured middle region (residues 373–412), which ensures the conformational flexibilities of the N- and C-terminal halves of zSec10 for their interdomain interaction.

Previous studies suggest that subcomplexes of the exocyst complex exist in mammalian and/or yeast cells. On the other hand, a recent electron microscopy study suggests that most of the exocyst subunits exist as the components of the entire exocyst complex in yeast^23^. Amino acid sequences of the exocyst subunits are well conserved among metazoans (*e.g.*, 36–84% identity between zSec10 and the Sec10 proteins from the representative metazoans shown in Fig. 2) but are somewhat different between fungi and metazoans (*e.g.*, 22% identity between yeast Sec10 and zSec10). This difference may affect the electrostatic and/or hydrophobic properties of the molecular surface of each subunit: yeast and mouse Exo70 structures are similar but substantially differ in surface electrostatic potential^18^, as mentioned above. Such difference might be related to the difference in regulation of the full or partial assembly of the exocyst subunits between yeast and mammals. The present structure of zSec10 does not show a biased distribution of hydrophobicity (Supplementary Fig. 3A). This property implies a weak assembly with other exocyst subunits to form a subcomplex.

Side-by-side helix–helix interactions have been found between Tip20 and Dsl1 in the Dsl1 complex, and between the SNARE protein Tlg1 and the Vps51 subunit of the GARP complex^33^,^43^. Similarly, the critical residues involved in the direct interaction between Myo2 and Sec15 were located on the sides of α-helices^44^, suggesting their side-by-side interaction. It seems that the side-by-side helix–helix interaction commonly occurs for the assembly or interaction of the CATCHR subunits and their structurally related proteins. Recent electron microscopy studies have suggested that the subunits of the COG, GARP and HOPS complexes are arranged in antiparallel, whereas those of the exocyst complex are arranged roughly in parallel^27^,^45^,^46^. Some or many of the inter-subunit interactions might be mediated by the side-by-side helix–helix interactions. Sec10, Exo70 and M-Sec (Sec6 homolog) exhibit a straight rod shape, whereas Tip20 exhibits a hooked rod shape, as mentioned above. The overall shapes of the CATCHR subunits may be diverse, although the structural information of their N-terminal halves is mostly unavailable. However, three structures of the exocyst subunit and its homolog adopt a similar straight structure (Fig. 4), which might allow the parallel arrangement of the exocyst subunits. The hooked or other undetermined shape of the N-terminal half structure might mediate the antiparallel subunit arrangement in other CATCHR complexes.

In conclusion, the crystal structure of near-full-length Sec10 retains its conserved α-solenoid architecture, which has been found in other CATCHR subunits. This structure provides a basis for further studies on its possible function as an individual molecule and/or as a subunit of the exocyst complex, and on its potential interactions with other proteins including other exocyst subunits, small GTPase and/or SNARE proteins. In particular, the present zSec10 structure will be useful for the interpretation of high-resolution three-dimensional images of the entire exocyst complex, which is expected to be determined in the near future.

## Materials and Methods

### Sample preparation

The gene encoding zSec10_195–708_ (Δ385–394) was amplified by PCR and cloned into the pGEX-6P-1 expression vector (GE Healthcare), using *Bam*HI and *Xho*I restriction sites to produce the N-terminally GST-fused protein. The protein was overproduced in *E. coli* Rosetta cells at 20 °C for 12–15 hours after induction with 0.1 mM isopropyl-β-D-thiogalactopyranoside (IPTG). The harvested cells were suspended in phosphate-buffered saline (PBS) containing 1 mM DTT and 0.1% Triton X-100 and lysed by sonication. The lysate was clarified by centrifugation and loaded onto a Glutathione Sepharose 4 Fast Flow column (GE Healthcare). The GST-fused protein was eluted with 50 mM Tris-HCl buffer (pH 8.0) containing 150 mM NaCl, 1 mM DTT and 15 mM reduced glutathione. The GST tag of the eluted protein was cleaved by PreScission protease (GE Healthcare). To remove the cleaved GST tag, protease and other minor impurities, the sample treated with protease was subjected to size-exclusion chromatography on a HiLoad 16/600 Superdex 200 column (GE Healthcare) with 10 mM Tris-HCl buffer (pH 7.5) containing 50 mM NaCl and 1 mM DTT. Finally, to completely remove the GST tag and GST-tagged protease, the sample was loaded onto a Glutathione Sepharose 4 Fast Flow column. The purified protein in the flowthrough fraction was collected and concentrated to 10 g L^−1^ using Amicon Ultra 15 (Millipore) for crystallization. The SeMet-substituted Sec10 was overexpressed in *E. coli* B834 cells at 20 °C for 24 hours after induction with 0.1 mM IPTG. The cells were grown in 200 mL culture supplemented with Core medium (Wako) containing all amino acids except methionine. Before induction with IPTG, 25 mg L^−1^ L-SeMet, 10 mg L^−1^ L-glucose and 250 mg L^−1^ MgSO_4_ were added. The SeMet-substituted zSec10_195–708_ (Δ385–394) was purified in the same manner as the native zSec10_195–708_ (Δ385–394).

### Limited proteolysis

Trypsin was mixed with the Sec10 samples at a weight ratio of 1:100. The mixtures were incubated for 12–36 hours at 4 °C. The protease-treated samples were fractionated by size-exclusion chromatography using a Superdex200 10/300 GL column with 10 mM Tris-HCl buffer (pH 7.5) containing 50 mM NaCl and 1 mM DTT. The peak fractions were analyzed by SDS-PAGE with Coomassie brilliant blue staining. For N-terminal amino acid sequencing, the protein bands were transferred to PVDF membranes (Immobilon-P, Merck Millipore) and analyzed using an ABI Procise Model 492 peptide sequencer.

### Crystallization

The native zSec10_195–708_ (Δ385–394) was crystallized at 20 °C by the hanging drop vapor diffusion method against a reservoir solution composed of 0.1 M HEPES-Na buffer (pH 7.5) and 0.57 M K Na tartrate after streak seeding with crushed native crystals as the seeds. The sample was mixed with the reservoir solution at a ratio of 1:1. The best rodlike crystals with dimensions of 0.25 × 0.05 × 0.05 mm^3^ appeared within 3–5 days. The seed crystals were obtained at 20 °C by the sitting drop vapor diffusion method against a reservoir solution composed of 0.1 M HEPES-Na buffer (pH 7.5) and 1 M K Na tartrate. The SeMet-substituted zSec10_195–708_ (Δ385–394) was crystallized against a reservoir solution composed of 0.1 M HEPES-Na buffer (pH 7.5) and 0.4 M K Na tartrate after streak seeding. The sample was mixed with the reservoir solution at a ratio of 1:0.6. The best SeMet-substituted protein crystals were grown with dimensions of 0.15 × 0.05 × 0.03 mm^3^. To increase the incorporation rate of SeMet, the crystallization process was repeated. The native crystals were used as the seeds for the first round of crystallization, and then the obtained SeMet-substituted protein crystals were used as the seeds for the second round.

### Data collection and structure determination

The obtained crystals were soaked in a 1:2 mixture of the reservoir solution and the saturated Li_2_SO_4_ solution containing 0.2 M NaBr and flash frozen by plunging into liquid nitrogen. The diffraction data sets of the native and SeMet-substituted protein crystals were collected at beamline BL41XU of SPring-8 (Hyogo, Japan) and beamline BL-17A of Photon Factory (Tsukuba, Japan), respectively. All diffraction data were processed using HKL2000 (HKL Research)^47^ and the CCP4 program suite^48^. To solve the structure from the 3.11-Å-resolution SAD data sets collected from the SeMet-substituted protein crystals, the program Phenix was used for heavy-atom site search, phase calculation and density modification^49^. Eighteen Se sites were identified. Phase extension using the 2.74-Å-resolution native data set was performed for automatic model building using the program Buccaneer (CCP4 package) with higher accuracy^50^. On the basis of the initial atomic model and the identified Se sites, the complete model of zSec10_195–708_ (Δ385–394) except residues 378–384 and 395–402 was built using the program Coot^51^. The structure was refined using the program Phenix. The final addition of 2 water molecules and 10 Br atoms decreased *R*_free_. In the final model, 94.7 % of the residues are in the most favored regions and 5.3 % are in the additional allowed regions. Data collection, phasing and refinement statistics are shown in Table 1. Electrostatic surface potential was calculated using the program APBS tool^52^. Structure figures were generated using the program PyMol (Delano Scientific; http://www.pymol.org) or CueMol (CueMol: Molecular Visualization Framework; http://www.cuemol.org). The protein surface color-coded according to hydrophobicity was drawn using the Python script “Color_h.py” for PyMol (downloaded from http://us.expasy.org/tools/pscale/Hphob.Eisenberg.html)^53^. Multiple sequence alignment was performed using the program ClustalW at EMBL-EBI to generate the alignment file in the ClustalW format^54^. With this alignment file, the surface conservation was calculated using the ConSurf server^55^. The protein surface color-coded according to sequence conservation was drawn using the Python script “consurf_new.py” in PyMol. The multiple-sequence-alignment figure was generated using ESPript 3.0^56^. The pairwise alignment of the structure of zSec10 with those of other CATCHR subunits and their structurally related proteins was performed using the Dali server^35^.

## Additional Information

**Accession codes:** Coordinates and structure factors of zSec10_195–708_ (Δ385–394) have been deposited in the Protein Data Bank under accession code 5H11.

**How to cite this article**: Chen, J. *et al*. Crystal structure of Sec10, a subunit of the exocyst complex. *Sci. Rep.* **7**, 40909; doi: 10.1038/srep40909 (2017).

**Publisher's note:** Springer Nature remains neutral with regard to jurisdictional claims in published maps and institutional affiliations.

## Supplementary Material

Supplementary Table and Figures

## Figures and Tables

**Table 1:** Data collection and refinement statistics.

	Native	SeMet
**Data collection**		
Beamline	SPring-8 BL41XU	PF BL-17A
Space group	*C*2	*C*2
Cell dimensions		
*a, b, c* (Å)	147.7, 162.7, 45.6	150.0, 161.3, 45.5
*α, β, γ* (˚)	90.0, 95.6, 90.0	90.0, 95.4, 90.0
Wavelength (Å)	0.9185	0.9788
Resolution (Å)	50.0–2.73 (2.78–2.73)	50.0–3.11 (3.16–3.11)
*R*_sym_ (%)	12.0 (38.4)	17.2 (45.2)
*I/σI*	10.7 (1.5)	14.5 (1.7)
Completeness (%)	95.3 (75.9)	94.6 (75.8)
Redundancy	8.2 (3.5)	29.8 (13.1)
**Refinement**		
Resolution (Å)	46.92–2.73 (2.83–2.73)	
No. reflections	26,989 (2,247)	
*R*_work_/*R*_free_ (%)	23.6/26.7 (34.1/37.4)	
Ramachandran plot		
favored, allowed, outliers (%)	94.7, 5.3, 0.0	
Clashscore	7.00	
No. atoms		
Protein	3,991	
Water	2	
Br	10	
*B*-factors (Å^2^)		
Protein	57.7	
Water	41.1	
Br	148.5	
R.m.s deviations		
Bond lengths (Å)	0.003	
Bond angles (˚)	0.61	

^*^Values in parentheses are for highest-resolution shell.

## References

[b1] Wu L. G., Hamid E., Shin W. & Chiang H. C. Exocytosis and endocytosis: modes, functions, and coupling mechanisms. Annu Rev Physiol 76, 301–331 (2014). 24274740 10.1146/annurev-physiol-021113-170305 PMC4880020

[b2] Wu B. & Guo W. The exocyst at a glance. J Cell Sci 128, 2957–2964 (2015). 26240175 10.1242/jcs.156398 PMC4541039

[b3] Novick P., Field C. & Schekman R. Identification of 23 complementation groups required for post-translational events in the yeast secretory pathway. Cell 21, 205–215 (1980). 6996832 10.1016/0092-8674(80)90128-2

[b4] TerBush D. R., Maurice T., Roth D. & Novick P. The Exocyst is a multiprotein complex required for exocytosis in *Saccharomyces cerevisiae*. EMBO J 15, 6483–6494 (1996). 8978675 PMC452473

[b5] Kee Y. . Subunit structure of the mammalian exocyst complex. Proc Natl Acad Sci USA 94, 14438–14443 (1997). 9405631 10.1073/pnas.94.26.14438 PMC25013

[b6] Zajac A., Sun X., Zhang J. & Guo W. Cyclical regulation of the exocyst and cell polarity determinants for polarized cell growth. Mol Biol Cell 16, 1500–1512 (2005). 15647373 10.1091/mbc.E04-10-0896 PMC551511

[b7] Liu J. & Guo W. The exocyst complex in exocytosis and cell migration. Protoplasma 249, 587–597 (2012). 21997494 10.1007/s00709-011-0330-1

[b8] Das A. & Guo W. Rabs and the exocyst in ciliogenesis, tubulogenesis and beyond. Trends Cell Biol 21, 383–386 (2011). 21550243 10.1016/j.tcb.2011.03.006 PMC3128673

[b9] Vega I. E. & Hsu S. C. The exocyst complex associates with microtubules to mediate vesicle targeting and neurite outgrowth. J Neurosci 21, 3839–3848 (2001). 11356872 10.1523/JNEUROSCI.21-11-03839.2001 PMC3674029

[b10] Bodemann B. O. . RalB and the exocyst mediate the cellular starvation response by direct activation of autophagosome assembly. Cell 144, 253–267 (2011). 21241894 10.1016/j.cell.2010.12.018 PMC3038590

[b11] Barlowe & Coupled ER to Golgi transport reconstituted with purified cytosolic proteins. J Cell Biol 139, 1097–1108 (1997). 9382859 10.1083/jcb.139.5.1097 PMC2140203

[b12] Yu I. M. & Hughson F. M. Tethering factors as organizers of intracellular vesicular traffic. Annu Rev Cell Dev Biol 26, 137–156 (2010). 19575650 10.1146/annurev.cellbio.042308.113327

[b13] Yamashita M. . Structural basis for the Rho- and phosphoinositide-dependent localization of the exocyst subunit Sec3. Nat Struct Mol Biol 17, 180–186 (2010). 20062059 10.1038/nsmb.1722

[b14] Baek K. . Structure-function study of the N-terminal domain of exocyst subunit Sec3. J Biol Chem 285, 10424–10433 (2010). 20139078 10.1074/jbc.M109.096966 PMC2856249

[b15] Fukai S., Matern H. T., Jagath J. R., Scheller R. H. & Brunger A. T. Structural basis of the interaction between RalA and Sec5, a subunit of the Sec6/8 complex. EMBO J 22, 3267–3278 (2003). 12839989 10.1093/emboj/cdg329 PMC165653

[b16] Sivaram M. V., Furgason M. L., Brewer D. N. & Munson M. The structure of the exocyst subunit Sec6p defines a conserved architecture with diverse roles. Nat Struct Mol Biol 13, 555–556 (2006). 16699513 10.1038/nsmb1096

[b17] Wu S., Mehta S. Q., Pichaud F., Bellen H. J. & Quiocho F. A. Sec15 interacts with Rab11 via a novel domain and affects Rab11 localization *in vivo*. Nat Struct Mol Biol 12, 879–885 (2005). 16155582 10.1038/nsmb987

[b18] Moore B. A., Robinson H. H. & Xu Z. The crystal structure of mouse Exo70 reveals unique features of the mammalian exocyst. J Mol Biol 371, 410–421 (2007). 17583731 10.1016/j.jmb.2007.05.018 PMC2692999

[b19] Dong G., Hutagalung A. H., Fu C., Novick P. & Reinisch K. M. The structures of exocyst subunit Exo70p and the Exo84p C-terminal domains reveal a common motif. Nat Struct Mol Biol 12, 1094–1100 (2005). 16249794 10.1038/nsmb1017

[b20] Jin R. . Exo84 and Sec5 are competitive regulatory Sec6/8 effectors to the RalA GTPase. EMBO J 24, 2064–2074 (2005). 15920473 10.1038/sj.emboj.7600699 PMC1150893

[b21] Zhang C. . Endosidin2 targets conserved exocyst complex subunit EXO70 to inhibit exocytosis. Proc Natl Acad Sci USA 113, E41–50 (2016). 26607451 10.1073/pnas.1521248112 PMC4711834

[b22] Croteau N. J., Furgason M. L., Devos D. & Munson M. Conservation of helical bundle structure between the exocyst subunits. PLoS One 4, e4443 (2009). 19214222 10.1371/journal.pone.0004443 PMC2635961

[b23] Heider M. R. . Subunit connectivity, assembly determinants and architecture of the yeast exocyst complex. Nat Struct Mol Biol (2015). 10.1038/nsmb.3146 PMC4752824 26656853

[b24] Katoh Y., Nozaki S., Hartanto D., Miyano R. & Nakayama K. Architectures of multisubunit complexes revealed by a visible immunoprecipitation assay using fluorescent fusion proteins. J Cell Sci 128, 2351–2362 (2015). 25964651 10.1242/jcs.168740

[b25] Lipschutz J. H. . Exocyst is involved in cystogenesis and tubulogenesis and acts by modulating synthesis and delivery of basolateral plasma membrane and secretory proteins. Mol Biol Cell 11, 4259–4275 (2000). 11102522 10.1091/mbc.11.12.4259 PMC15071

[b26] Roth D., Guo W. & Novick P. Dominant negative alleles of SEC10 reveal distinct domains involved in secretion and morphogenesis in yeast. Mol Biol Cell 9, 1725–1739 (1998). 9658167 10.1091/mbc.9.7.1725 PMC25411

[b27] Prigent M. . ARF6 controls post-endocytic recycling through its downstream exocyst complex effector. J Cell Biol 163, 1111–1121 (2003). 14662749 10.1083/jcb.200305029 PMC2173613

[b28] D’Souza-Schorey C., Li G., Colombo M. I. & Stahl P. D. A regulatory role for ARF6 in receptor-mediated endocytosis. Science 267, 1175–1178 (1995). 7855600 10.1126/science.7855600

[b29] Fielding A. B. . Rab11-FIP3 and FIP4 interact with Arf6 and the exocyst to control membrane traffic in cytokinesis. EMBO J 24, 3389–3399 (2005). 16148947 10.1038/sj.emboj.7600803 PMC1276165

[b30] Zuo X., Guo W. & Lipschutz J. H. The exocyst protein Sec10 is necessary for primary ciliogenesis and cystogenesis *in vitro*. Mol Biol Cell 20, 2522–2529 (2009). 19297529 10.1091/mbc.E08-07-0772 PMC2682593

[b31] Ward J. J., Sodhi J. S., McGuffin L. J., Buxton B. F. & Jones D. T. Prediction and functional analysis of native disorder in proteins from the three kingdoms of life. J Mol Biol 337, 635–645 (2004). 15019783 10.1016/j.jmb.2004.02.002

[b32] Richardson B. C. . Structural basis for a human glycosylation disorder caused by mutation of the COG4 gene. Proc Natl Acad Sci USA 106, 13329–13334 (2009). 19651599 10.1073/pnas.0901966106 PMC2716380

[b33] Tripathi A., Ren Y., Jeffrey P. D. & Hughson F. M. Structural characterization of Tip20p and Dsl1p, subunits of the Dsl1p vesicle tethering complex. Nat Struct Mol Biol 16, 114–123 (2009). 19151722 10.1038/nsmb.1548 PMC2635920

[b34] Perez-Victoria F. J. . Structural basis for the wobbler mouse neurodegenerative disorder caused by mutation in the Vps54 subunit of the GARP complex. Proc Natl Acad Sci USA 107, 12860–12865 (2010). 20615984 10.1073/pnas.1004756107 PMC2919957

[b35] Holm L. & Rosenstrom P. Dali server: conservation mapping in 3D. Nucleic Acids Res 38, W545–549 (2010). 20457744 10.1093/nar/gkq366 PMC2896194

[b36] Kimura S. . Distinct Roles for the N- and C-terminal regions of M-Sec in plasma membrane deformation during tunneling nanotube formation. Sci Rep 6, 33548 (2016). 27629377 10.1038/srep33548 PMC5024327

[b37] Velvarska H. & Niessing D. Structural insights into the globular tails of the human type V myosins Myo5a, Myo5b, And Myo5c. PLoS One 8, e82065 (2013). 24339992 10.1371/journal.pone.0082065 PMC3858360

[b38] Li W. . The crystal structure of a Munc13 C-terminal module exhibits a remarkable similarity to vesicle tethering factors. Structure 19, 1443–1455 (2011). 22000513 10.1016/j.str.2011.07.012 PMC3197213

[b39] Munson M., Chen X., Cocina A. E., Schultz S. M. & Hughson F. M. Interactions within the yeast t-SNARE Sso1p that control SNARE complex assembly. Nat Struct Biol 7, 894–902 (2000). 11017200 10.1038/79659

[b40] He B., Xi F., Zhang X., Zhang J. & Guo W. Exo70 interacts with phospholipids and mediates the targeting of the exocyst to the plasma membrane. EMBO J 26, 4053–4065 (2007). 17717527 10.1038/sj.emboj.7601834 PMC2230670

[b41] Songer J. A. & Munson M. Sec6p anchors the assembled exocyst complex at sites of secretion. Mol Biol Cell 20, 973–982 (2009). 19073882 10.1091/mbc.E08-09-0968 PMC2633393

[b42] Papaleo E. . The role of protein loops and linkers in conformational dynamics and allostery. Chem. Rev. 116, 6391–6423 (2016). 26889708 10.1021/acs.chemrev.5b00623

[b43] Fridmann-Sirkis Y., Kent H. M., Lewis M. J., Evans P. R. & Pelham H. R. Structural analysis of the interaction between the SNARE Tlg1 and Vps51. Traffic 7, 182–190 (2006). 16420526 10.1111/j.1600-0854.2005.00374.x

[b44] Jin Y. . Myosin V transports secretory vesicles via a Rab GTPase cascade and interaction with the exocyst complex. Dev Cell 21, 1156–1170 (2011). 22172676 10.1016/j.devcel.2011.10.009 PMC3241923

[b45] Ha J. Y. . Molecular architecture of the complete COG tethering complex. Nat Struct Mol Biol 23, 758–760 (2016). 27428773 10.1038/nsmb.3263 PMC4972656

[b46] Chou H. T., Dukovski D., Chambers M. G., Reinisch K. M. & Walz T. CATCHR, HOPS and CORVET tethering complexes share a similar architecture. Nat Struct Mol Biol 23, 761–763 (2016). 27428774 10.1038/nsmb.3264 PMC4972687

[b47] Otwinowski Z. & Minor W. Processing of X-ray diffraction data collected in oscillation mode. Methods Enzymol part A 276, 307–326 (1997). 10.1016/S0076-6879(97)76066-X 27754618

[b48] Winn M. D. . Overview of the CCP4 suite and current developments. Acta Crystallogr D 67, 235–242 (2011). 21460441 10.1107/S0907444910045749 PMC3069738

[b49] Adams P. D. . PHENIX: a comprehensive Python-based system for macromolecular structure solution. Acta Crystallogr D 66, 213–221 (2010). 20124702 10.1107/S0907444909052925 PMC2815670

[b50] Cowtan K. The Buccaneer software for automated model building. 1. Tracing protein chains. Acta Crystallogr D 62, 1002–1011 (2006). 16929101 10.1107/S0907444906022116

[b51] Emsley P., Lohkamp B., Scott W. G. & Cowtan K. Features and development of Coot. Acta Crystallogr D 66, 486–501 (2010). 20383002 10.1107/S0907444910007493 PMC2852313

[b52] Baker N. A., Sept D., Joseph S., Holst M. J. & McCammon J. A. Electrostatics of nanosystems: application to microtubules and the ribosome. Proc Natl Acad Sci USA 98, 10037–10041 (2001). 11517324 10.1073/pnas.181342398 PMC56910

[b53] Eisenberg D., Schwarz E., Komaromy M. & Wall R. Analysis of membrane and surface protein sequences with the hydrophobic moment plot. J Mol Biol 179, 125–142 (1984). 6502707 10.1016/0022-2836(84)90309-7

[b54] Larkin M. A. . Clustal W and Clustal X version 2.0. Bioinformatics 23, 2947–2948 (2007). 17846036 10.1093/bioinformatics/btm404

[b55] Ashkenazy H. . ConSurf 2016: an improved methodology to estimate and visualize evolutionary conservation in macromolecules. Nucleic Acids Res 44, W344–W350 (2016). 27166375 10.1093/nar/gkw408 PMC4987940

[b56] Robert X. & Gouet P. Deciphering key features in protein structures with the new ENDscript server. Nucleic Acids Res 42, W320–324 (2014). 24753421 10.1093/nar/gku316 PMC4086106

