# Supplementary material for: Crystal structure of Sec10, a subunit of the exocyst complex
Source: Sci Rep. 2017 Jan 18;7:40909. doi: 10.1038/srep40909 (PMC5241887; doi:10.1038/srep40909)
Supplement: Supplementary Table and Figures [file srep40909-s1.pdf]

Supplementary Information

Crystal structure of Sec10, a subunit of the exocyst complex

Jianxing Chen[1,2,3], Atsushi Yamagata[1,2,3], Keiko Kubota[1], Yusuke Sato[1,2,3], Sakurako Goto-Ito[1,2,3], Shuya Fukai[1,2,3]

[1]Structural Biology Laboratory, Structural Life Science Division, Synchrotron Radiation Research Organization and Institute of Molecular and Cellular Biosciences, The University of Tokyo, Tokyo 113-0032, Japan

[2]Department of Computational Biology and Medical Sciences, Graduate School of Frontier Sciences, The University of Tokyo, Chiba 277-8501, Japan

[3]CREST, JST, Saitama 332-0012, Japan

Correspondence should be addressed to S.F. (fukai@iam.u-tokyo.ac.jp).

Expression construct	Soluble expression level	Crystallization
**$rSec10_{88\text{-}351}$**	**low**	**no**
**$rSec10_{129\text{-}351}$**	**high**	**no**
**$rSec10_{378\text{-}708}$**	**low**	**no**
**$rSec10_{402\text{-}708}$**	**low**	**no**
**$rSec10_{412\text{-}708}$**	**low**	**no**
**$rSec10_{421\text{-}708}$**	**low**	**no**
**$rSec10_{114\text{-}708}$**	**high**	**yes**
**$rSec10_{114\text{-}708}$ (Δ368-412)**	**high**	**no**
**$rSec10_{114\text{-}708}$ (Δ368-393)**	**high**	**no**
**$rSec10_{114\text{-}708}$ (Δ378-412)**	**high**	**no**
**$rSec10_{114\text{-}708}$ (Δ378-393)**	**high**	**yes**
**$rSec10_{114\text{-}708}$ (Δ385-395)**	**high**	**yes**
**$rSec10_{114\text{-}708}$ (Δ378-395)**	**high**	**yes**
**$zSec10_{114\text{-}708}$ (Δ378-395)**	**high**	**no**
**$zSec10_{114\text{-}708}$ (Δ378-394)**	**high**	**yes**
**$zSec10_{114\text{-}708}$ (Δ385-394)**	**high**	**yes**
**$zSec10_{114\text{-}708}$ (Δ386-392)**	**high**	**no**
**$zSec10_{114\text{-}708}$ (Δ385-393)**	**high**	**no**
**$zSec10_{114\text{-}708}$ (Δ386-389)**	**high**	**yes**
**$zSec10_{114\text{-}708}$ (Δ390-392)**	**high**	**yes**
**$zSec10_{114\text{-}708}$ (Δ390)**	**high**	**no**
**$zSec10_{114\text{-}708}$ (Δ392)**	**high**	**no**
**$zSec10_{114\text{-}708}$ (Δ394)**	**high**	**no**
**$zSec10_{114\text{-}708}$ (R390A)**	**high**	**no**
**$zSec10_{127\text{-}708}$ (Δ385-394)**	**high**	**no**
**$zSec10_{147\text{-}708}$ (Δ385-394)**	**high**	**yes**
**$zSec10_{189\text{-}708}$ (Δ385-394)**	**high**	**no**
**$zSec10_{195\text{-}708}$ (Δ385-394)**	**high**	**yes**

Supplementary Table 1

List of designed expression constructs of rSec10 and zSec10

These expression constructs are designed on the basis of computationally and experimentally predicted disordered regions. Their soluble expression level and crystallization property are defined qualitatively as low/high and yes/no, respectively.

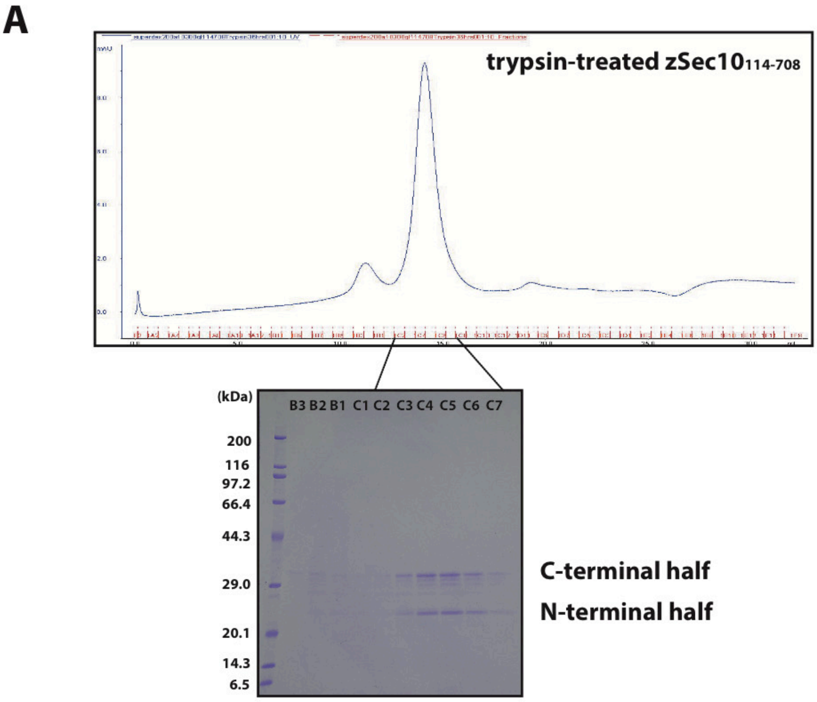

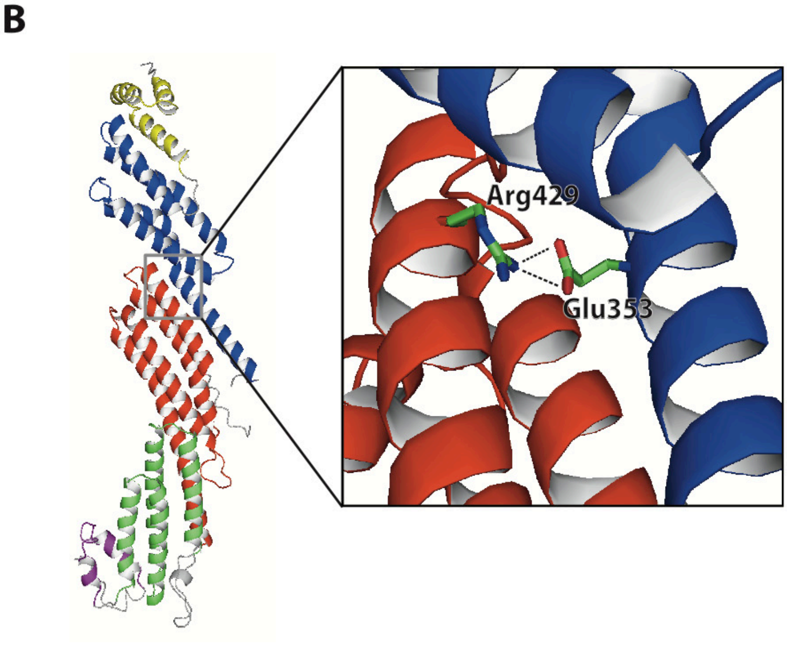


```
D. rerio          YLESYIDMERQYLQNRSG----......TRHAFERCNKLS-------------DPAD
H. sapiens        YLENYIEVETGYLKSRSA----......TKQAFERCHRLS-------------DPSD
M. musculus       YLENYIEVEIGYLKSRSA----......TKQAFERCHRLS-------------DPSD
R. norvegicus     YLENYIEVEIGYLKSRSA----......TKQAFERCHRLS-------------DPSD
G. gallus         YLENYIEVEIGYLKSRSA----......TKQAFERCHRLS-------------DPSD
D. melanogaster   HLAGYAEMETKCLTAKCS----......AKASLKRCRLLS-------------NETE
C. elegans        YIATYCSEELKYLNDQCS----......TKNAFGRANQLC-------------DKEE
S. cerevisiae     LFSHYLYDRSKYFGIEKRSLEA......ANYSLNDVDSMLKCVVESTARVMELIPNK
A. thaliana       HKDEYPEHERASLKQLYQ----......PATLAANVKAIFTCLLDQVS-VYITEGLE
```

Supplementary Figure 1 Interaction between N- and C-terminal halves of Sec10

(**A**) Elution profile of the size-exclusion chromatography of the trypsin-treated zSec10$_{114\text{-}708}$. The peak fractions were analyzed by SDS-PAGE. Two polypeptides corresponding to the N- and C-terminal halves were co-eluted as a single peak.

(**B**) Hydrogen bonds between Glu353 on helix H7 and Arg429 on helix H8 in zSec10. Both residues are shown as sticks. The hydrogen bonds are indicated as dotted lines. These two residues are conserved among metazoans.

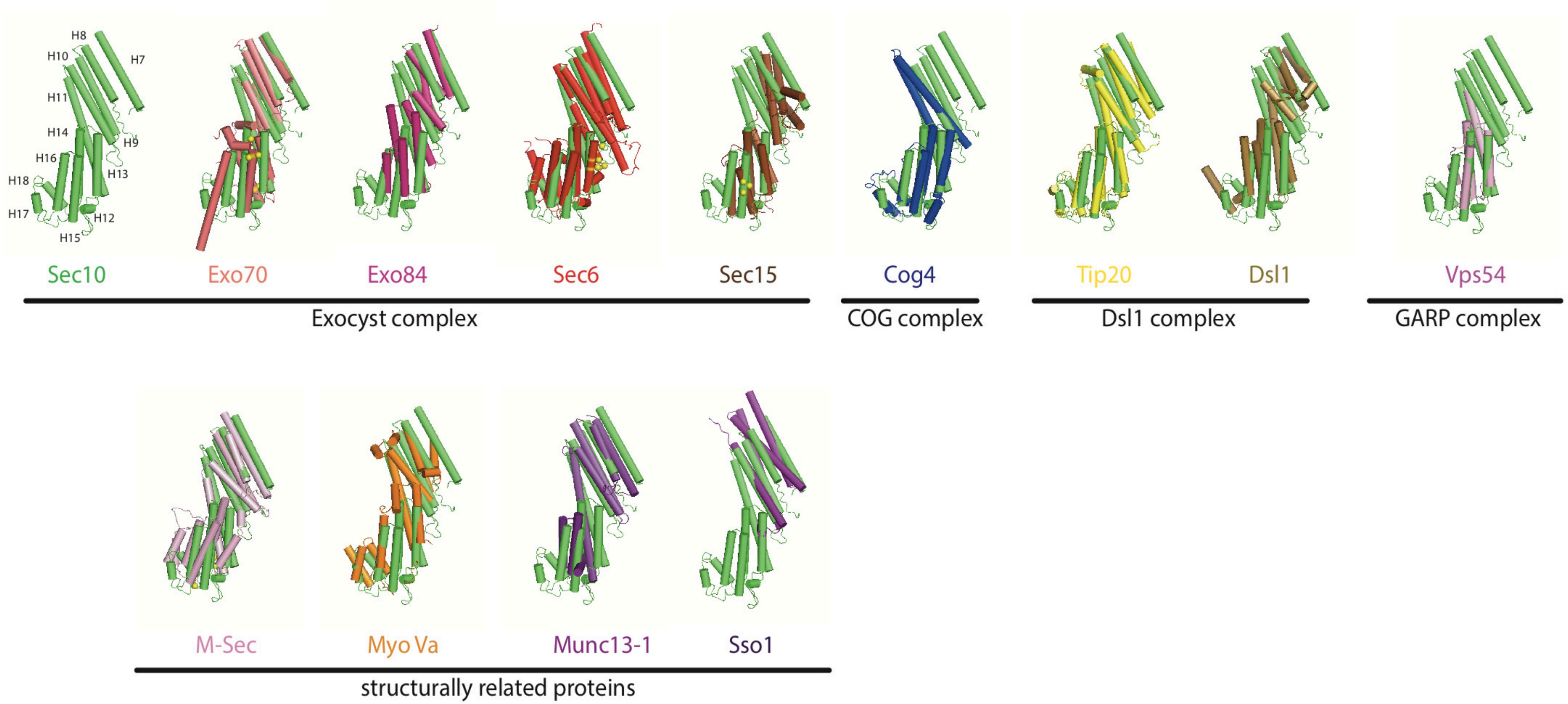

Supplementary Figure 2
(**A, B**) zSec10 C-terminal half containing domains C, D and E superposed with other determined CATCHR complex subunits and structurally related proteins. The function-related residues identified in a previous study are shown as yellow spheres for *S. cerevisiae* Exo70, *S. cerevisiae* Sec6, *D. melanogaster* Sec15 and *M. musculus* M-Sec.

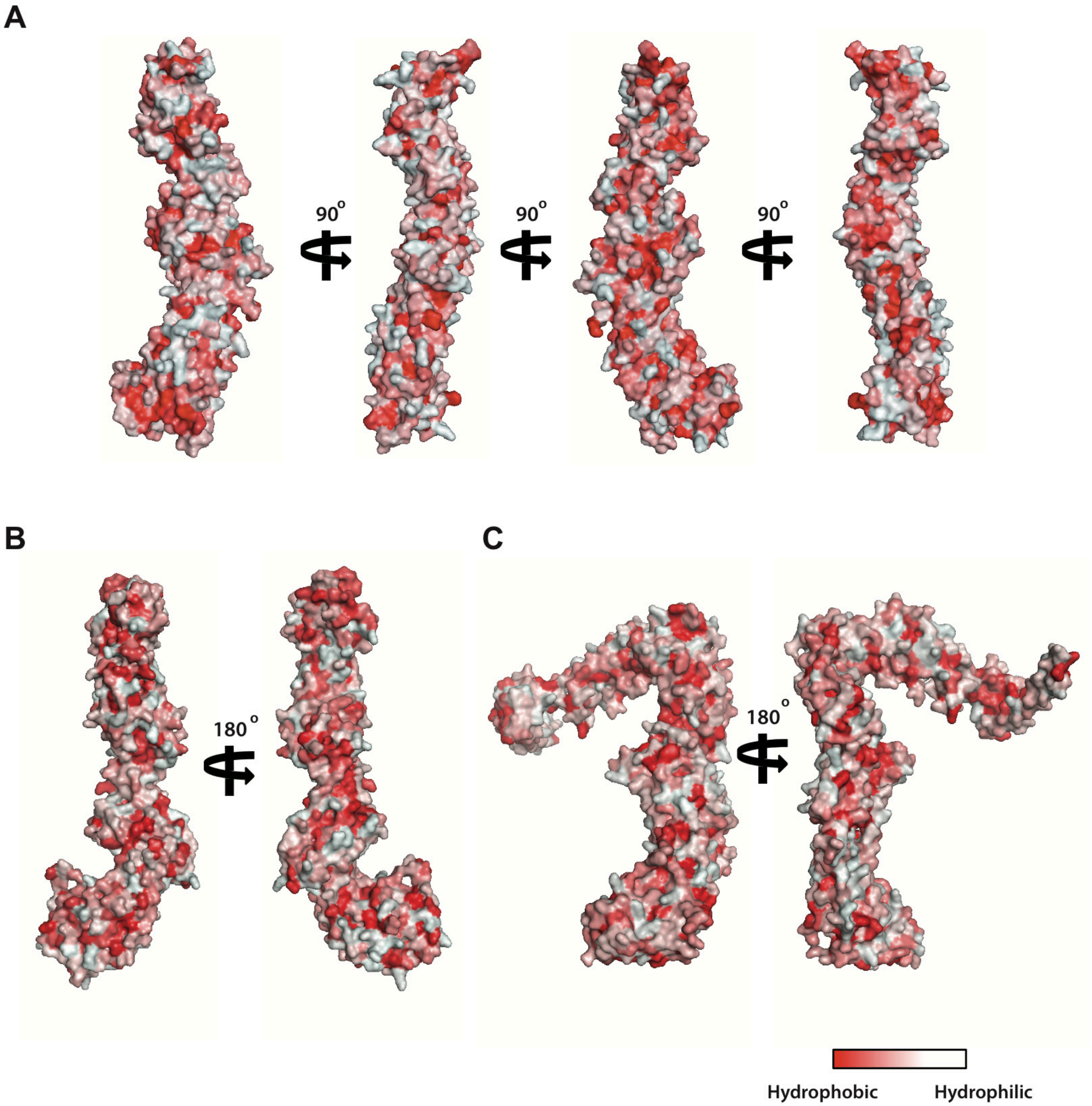

Supplementary Figure 3

Surface hydrophobicity distribution

(**A**) The zSec10 structure is colored according to its surface hydrophobicity, in which the hydrophobic region tends to be red and the hydrophilic region tends to be in white. zSec10 shows no polarity of hydrophobicity distribution as predicted in *S. cerevisiae* Sec10.

(**B, C**) *M. musculus* M-sec (B) and *S. cerevisiae* Tip20 (C) both show no biased distribution of hydrophobicity on their surface as well as zSec10.
